# New Insight from Using Spatiotemporal Image Correlation in Prenatal Screening of Fetal Conotruncal Defects

**Published:** 2013-09-18

**Authors:** Zuo-ping Xie, Bo-wen Zhao, Hua Yuan, Qi-qi Hua, She-hong Jin, Xiao-yan Shen, Xin-hong Han, Jia-mei Zhou, Min Fang, Jin-hong Chen

**Affiliations:** 1 Department of Diagnostic Ultrasound, Shaoxing Women and Children’s Hospital, Shaoxing, Zhejiang, China; 2 Department of Diagnostic Ultrasound and Echocardiography, Sir Run Run Shaw Hospital, Zhejiang University College of Medicine, Hangzhou, China

**Keywords:** Spatiotemporal Image Correlation, Ultrasonography, Fetus, Conotruncal Defects

## Abstract

**Background:**

To establish the reference range of the angle between ascending aorta and main pulmonary artery of fetus in the second and third trimester using spatiotemporal image correlation (STIC), and to investigate the value of this angle in prenatal screening of conotruncal defects (CTDs).

**Materials and Methods:**

Volume images of 311 normal fetuses along with 20 fetuses with congenital heart diseases were recruited in this cross-sectional study. An offline analysis of acquired volume datasets was carried out with multiplanar mode. The angle between aorta and pulmonary artery was measured by navigating the pivot point and rotating axes and the reference range was established. The images of ascending aorta and main pulmonary artery in fetuses with congenital heart diseases were observed by rotating the axes within the normal angle reference range.

**Results:**

The angle between ascending aorta and main pulmonary artery of the normal fetus (range: 59.1˚~97.0˚, mean ± SD: 78.0˚ ± 9.7˚) was negatively correlated with gestational age (r = -0.52; p<0.01). By rotating the normal angle range corresponding to gestational age, the fetuses with CTD could not display views of their left ventricular long axis and main pulmonary trunk correctly.

**Conclusion:**

The left ventricular long axis and main pulmonary trunk views can be displayed using STIC so that the echocardiographic protocol of the cardiovascular joint could be standardized. The reference range of the angle between ascending aorta and main pulmonary artery is clinically useful in prenatal screening of CTD and provides a reliable quantitative standard to estimate the spatial relationship of the large arteries of fetus.

## Introduction

During the past three decades, technical improvements in two dimensional (2D) gray scale, with the advent of transducers mounted with color and pulsed Doppler capabilities, has enabled an earlier prenatal diagnosis of fetal cardiac anomalies (1). Unfortunately, due to difficulties in defining the spatial relationship of the great arteries, prenatal diagnosis of conotruncal defects (CTDs) still represents the most challenging area in the field of fetal echocardiography (2). Let alone the routine fetal ultrasound evaluations, CTDs were more likely to be misdiagnosed or undetected until birth than other categories of structural heart defects (3).

Spatiotemporal image correlation (STIC) is a four dimensional (4D) ultrasound technology that allows analysis of the image data according to the spatial and temporal domain, and processes an offline dynamic three-dimensional (3D) image sequence after an automatic volume scan. STIC can provide a standardized display of cardiac anatomy, which may reduce the operator dependency in the diagnosis of congenital heart disease (4-7), and can also visualize the relationships, sizes, and courses of the outflow tracts (8, 9). Therefore, STIC is able to help the examiner to better understand the spatial relationship of the great vessels.

The object of this study is to establish the reference range of the angle between ascending aorta and main pulmonary artery of fetus in the second and third trimester using STIC, and to investigate the value of this angle in prenatal screening of CTD.

## Materials and Methods

The study was approved by the Clinical Ethics Committee of the Shaoxing Women and Children’s Hospital. From August 2008 to October 2009, all pregnant women undergoing fetal echocardiography were invited to participate in this cross-sectional study. Inclusion criteria were: 1. All women were nonsmokers and had no history of illicit drug consumption. 2. Fetal age was determined by the first day of the last menstrual period and was confirmed by first-trimester and early second-trimester sonographic measurements. 3. Volume datasets were obtained with the STIC technique. 4. Diagnoses were confirmed either after birth or by necroscopy. 5. Participants Signed Institutional Review Board-approved consent forms. Exclusion criteria included: 1. Unwilling to offer consent forms and to participate in this investigation. 2. Poor STIC volume datasets. 3. Confirmed fetal structural malformations of other systems.

To collect the STIC volume datasets, a Voluson 730 Expert (Ultrasound Unit) (GE Healthcare, Milwaukee, WI, USA) was used with a 4-8 MHz transabdominal volume transducer. The STIC volume was sampled at the level of the apical four-chamber view with the volume scan angle of 30-40 degrees and the acquisition time of 10-12.5 seconds. Volume datasets with the following characteristics were considered to be of high quality: 1. The preferred fetal position was with the sternum toward the transducer and the spine away from the transducer. 2. Minimal or no motion artifact was observed in the sagittal plane. Volume dataset acquisition was repeated if necessary to achieve three high-quality images.

**Fig 1:**
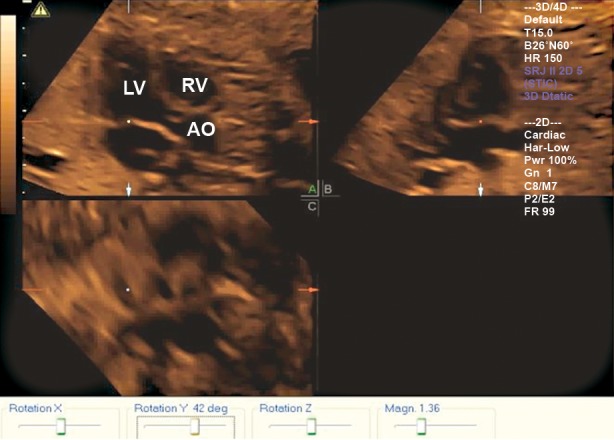
A plane is displayed in a normal fetus of 24 gestational weeks by rotating standard four chamber view of Y-axis clockwise, which shows LV long-axis view. LV; Left ventricular, Ao; Aorta and RV; Right ventricular.

**Fig 2:**
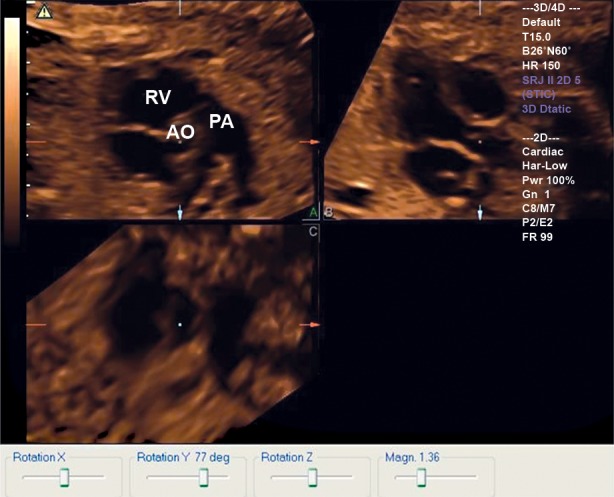
Based on figure 1, applying orthogonal point to the upper of the aortic valve, then rotating Y-axis clockwise about 77 degree , the main pulmonary trunk view is displayed at A plane. RV; Right ventricular, Pa; Pulmonary artery and Ao; Aorta.

The eligible STIC volume data were reformatted into orthogonal planes (panels A: midsagittal, panel B: axial and panels C coronal). The volume data were analyzed using Voluson 4D View 9.0 postprocessing software (GE Healthcare, Milwaukee, WI, USA). Angle between ascending aorta and main pulmonary artery (AAPA) was acquired as frame of end diastolic volume, when the mitral and tricuspid valves were closed by the frame-to-frame analysis of the virtual cardiac cycle of 4D STIC. The volume datasets were adjusted to the 4-chamber view in panel A. The reference dot was then positioned on the center of the mitral valve. The image was rotated on the Z-axis to display the apex of the left ventricle positioned at 12 o’clock and rotated on the Y-axis until the left ventricle outflow was displayed (Fig 1). The reference dot was then placed above the aortic valve, and the angle between ascending aorta and main pulmonary artery was measured by clicking in panel A, scrolling the Y-axis to the right until the short axis of the heart was visualized in panel A (Fig 2). The angle displayed in the Rotation Y option was then recorded. The relationship between the angle and gestational age was assessed by correlation and regression analysis and the reference range was established.

For fetuses with congenital heart diseases, by rotating the axes within the normal angle reference range, which were established in this study to determine if the images of ascending aorta and main pulmonary artery could be obtained.

To determine intra-observer variability, the initial observer re-measured the angles in 20 randomly selected fetuses. To evaluate the inter-observer variability, the angles in 20 randomly selected fetuses were analyzed by two independent observers, each of whom was blind to the results obtained by the other. Variability was presented as a mean percentage error (absolute difference divided by the average of the two observations).

### Statistical analysis

All data were analyzed as the mean value ± SD. All initial data were tested for normality using Kolmogorov-Smirnov test. Scatter diagram of AAPA and gestation weeks was analyzed by curvilinear regression. The mean value of AAPA was expressed as χ ± s, and 95% confidence interval was estimated; A p value <0.05 was considered to be statistically significant. All analyses were carried out using the statistical package for the social sciences (SPSS) software version 16.0 (SPSS Inc., Chicago, IL, USA)

## Results

**Fig 3:**
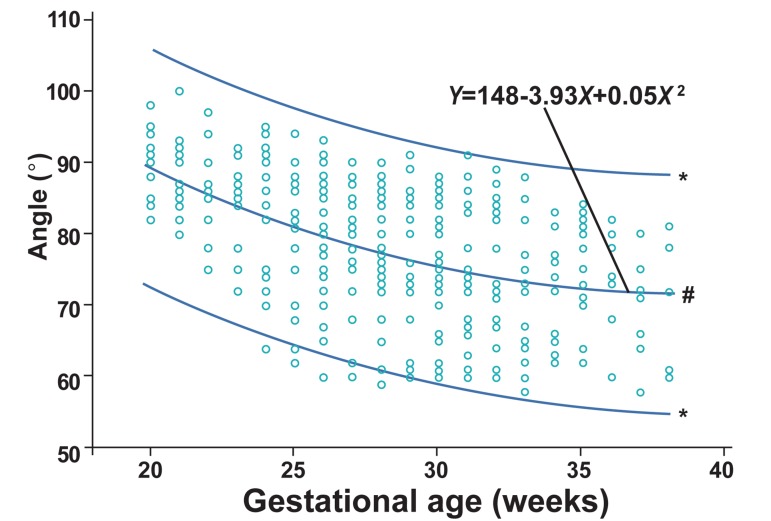
Scatterplot distribution and fitting curve of the angle between the aorta and pulmonary artery (AAPA). #; Fitting Curve of AAPA and gestational weeks and *; 95% CI of AAPA.

STIC volume acquisition was successfully obtained for a total of 331 women. Of these, 28 normal fetuses and 1 fetus with congenital heart disease(CHD) were later excluded because of poor volume datasets. The AAPA was successfully collected in 302 (91.2%) cases in which 283 were normal fetuses while 19 were cases with CHD. The angle ranging from 59 to 97.0 degrees was negatively correlated with gestational age [correlation coefficient (r):-0.52; p<0.01]. The best-fit exponential curve regression equation of the angle was Y=148-3.93X + 0.05X2 (Fig 3). 

**Fig 4:**
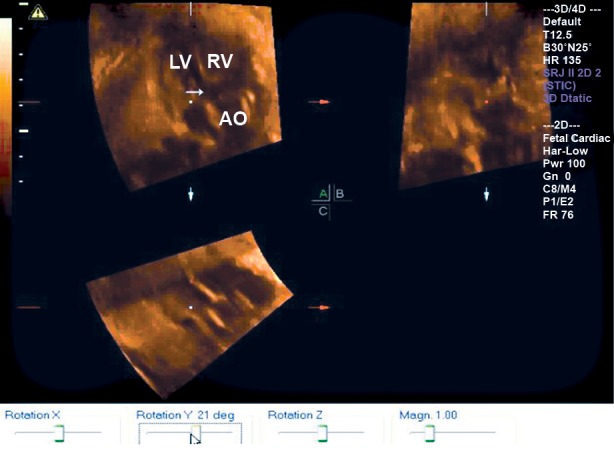
STIC in A plane shows a wide aorta overriding the VSD of a 27-week fetus diagnosed with Tetralogy of Fallot. LV; left ventricular, RV; Right ventricular, Ao; Aorta and →; Ventricular septal defect.

Nineteen cases of CHD were divided into two groups, 9 in CTD group and 10 in non-CTD group, according to the neonate echocardiography or necroscopy. By rotating the normal angle range corresponding to gestational age, the fetuses with CTD could not display their left ventricular long axis and main pulmonary trunk views correctly (Fig 4). Two cases of perimembranous-ventricular septal defect (VSD) and one case of aortic stenosis with VSD did not show the normal left ventricular long axis views in the non-CTD group. Ten cases in the nonCTD group showed the normal main pulmonary trunk view (Table 1).

Inter-and intraobserver variability percentage was determined to be 13.6 ± 3.1 and 8.7 ± 1.4 respectively

**Table 1:** Details of 19 fetuses of STIC displaying LV long axis and main pulmonary trunk views and postnatal diagnosis in 19 CHD fetus


No.	GW (wks)	LV long-axis view display	MPT view display	Postnatal diagnosis

**1**	27^+^^6^	Display mitral valve-LV outflow-Ascending Aorta view,broad Aorta overriding over VSD	no-display	Tetralogy of Fallot, VSD^Δ^
**2**	25^+^^6^	ditto	no-display	Tetralogy of Fallot, VSD^Δ^
**3**	30^+^^1^	ditto	no-display	Tetralogy of Fallot, VSD^Δ^
**4**	36^+^^1^	ditto	no-display	Tetralogy of Fallot, VSD^Δ^
**5**	24^+^^4^	Display mitral valve-LV outflow- ascending Aorta view, while the aorta overriding VSD, which most originated from RV	no-display	Double Outlet Right Ventricle, VSD, LV Dysplasia^Δ^
6*	25^+^^3^	no-display	no-display	Mitral atresia, Double Outlet Right Ventricle, Aortic Arch and Ascending Aorta Dysplasia.
**7**	38^+^^5^	Display mitral valve-LV outflow-Ascending Aorta view, broad Aorta overriding over VSD	no-display	Arteriosus, VSD
**8**	26^+^^5^	Display mitral valve-LV outflow-Ascending Aorta view,broad Aorta overriding over VSD	no-display	Persistent truncus arteriosus, VSD^Δ^
**9**	27^+^^4^	Display mitral valve-LV outflow-Ascending Aorta view, A main artery originated from LV extends a short distance and shows forklike.	no-display	Transposition of the great arteries, VSD.
**10**	32	Display mitral valve-LV outflow-Ascending Aorta view, there exist discontinuity between Aorta and interventricular septum.	normal-display	Perimembranous VSD
**11**	36^+^^3^	ditto	normal-display	Perimembranous VSD
**12**	27	normal-display	normal-display	Muscular VSD
**13**	28^+^^2^	normal-display	normal-display	Muscular VSD
**14**	26^+^^6^	normal-display	normal-display	Muscular VSD
**15**	27^+^^4^	Display normal mitral valve-LV outflow-ascending aorta view,there exist discontinuity between Ascending Aorta stenosis and interventricular septum.	normal-display	Aortic Stenosis, VSD^Δ^
**16**	28^+^^5^	normal-display	normal-display	Ostium primum ASD
**17**	34	normal-display	normal-display	Endocardial Cushion Defects transition form.^Δ^
**18**	31	normal-display	normal-displav	Ebtein’s Anomaly
**19**	28^+^^3^	normal-display	normal-display	Cardiac Rhabdomyoma


## Discussion

The right ventricular outflow (RVO) originated from the right and anterior to the left ventricular outflow (LVO), and extended to the posterior and the left. LVO went to right at the level of aortic valve. RVO located to the left side of LVO and sent out to main pulmonary artery, which made pulmonary valves situate on the left and anterior to aortic valves. Main pulmonary artery continually extended to the right and posterior, and divided into left and right pulmonary arteries between the rear of ascending aorta and below of the aortic arch (10, 11). Therefore, an angle presented between the left ventricular long axis view and RVO-main pulmonary trunk-pulmonary artery cross view. CTD developed from the obstruction or arrest of cone truncus arteriosis growth during embryogenesis. Cone septum agenesia or malrotation destroyed the proper fusion of the interventricular septum muscular part, which attributed to the abnormal change of AAPA.

The 3D data of fetal heart was acquired by STIC technique in this study. Applying orthogonal multiplanar mode, adjusting the position of orthogonal point and rotating X-Y-Z-axis, we obtained left ventricular long axis view (mitral valve-left ventricular outflow-ascending aorta view) and main pulmonary trunk view (right ventricular outflow-main pulmonary trunk-pulmonary artery cross view). It made the display of the conjunction part of main artery more quantitated and programmed, and decreased the dependence on operator subjectivity. The measurement of AAPA could be able to make a quantitative analysis of the spatial relationship of fetal heart great arteries by echocardiography.

Espinoza et al. found a correlation between fetal heart main arteries spatial angle and gestation week in 85 cases using STIC (12). Based on the statistical analysis of the large sample size, we established the normal range of AAPA reference values. In this study, we chose the frame of end diastolic volume as the standard plane to measure the AAPA, due to following reasons: 1) mitral valves and aortic valves were off state in this phase, which may help to locate the orthogonal point and minimize the error of measurement: 2) the angles between the views of main arteries would change accordingly because of the heart beat. If we select the preferred view, the results would remain reliable. Our data also showed an inverse correlation between the AAPA and advancing gestational weeks. The conotruncal septum has developed its rotation since the 31^th^ or 32^rd^ day of embryogenesis, and the spatial relation between aorta and pulmonary artery has shaped (13). With the development of human embryo, AAPA would change accompanied by the upgrowth of cardiovascular system and adjacent organs such as lung and liver (14). This could explain the association of AAPA changing with the increase of gestational weeks. Our study indicated that the slow downtrend of AAPA with the increase of gestation weeks may reflect the AAPA being stabilized as the maturity of fetal heart.

We failed to obtain the LV long-axis and main pulmonary trunk views in 19 CHD cases using STIC derived AAPA reference ranges as we did in normal fetuses. This finding indicates that the establishment of normal reference range of AAPA can offer the objective reference standards in the screening of fetal heart by STIC. For fetuses that did not display normal LV long-axis and main pulmonary trunk views using novel STIC derived AAPA , a high possibility of conotruncal arteriosis deformity is suggested, therefore, the detailed fetal echocardiographic evaluation was recommended.

Two cases of perimembranous VSD showed the discontinuation between aorta and interventricular septum in left ventricular long-axis view. It was hard to determine whether the aorta overrode ventricular septum or not, because of the large deletion in ventricular septum. Continuing to roll the transducer, we can acquire the main pulmonary view. Therefore, the AAPA may identify the VSD with conotruncal defects in fetal heart ultrasonography. When it is difficult to identify whether the VSD is combined with CTD, AAPA would help us to make the right decision.

Nevertheless, several limitations of this study need to be addressed. First, the cross-sectional design may reduce the power of observed correlations. Second, as the limitation of 2D ultrasound imaging, the quality of 3D STIC reconstructed image could also be interfered by respiration movement of gravida, random movement of fetus, fast and irregular fetal heart beat, and the block of ribs or spinal column acoustic shadows. Third, for 8.7% of fetuses (29/331) volume data could not be obtained satisfactorily. Forth, volume images collected by one examination may not be sufficient to cover all the important information for analysis of fetus over 20 gestational weeks, while frequent fetal movement may disturb the acquirement of image for fetus below 20 gestational weeks. Fifth, in this investigation we could not get exact angle between ascending aorta and main pulmonary artery in fetuses with CTD as we did in normal fetuses, therefore we did not compare the angles of normal fetuses with those with CTD, but the normal angle reference range established was used in this study to determine if the images of ascending aorta and main pulmonary artery could be obtained by rotating the axes within the normal AAPA. Finally, the reproducibility test showed that the discrepancy rate was 13.6% in our study, which suggested the observer-dependent nature of this measurement.

## Conclusion

The left ventricular long axis and main pulmonary trunk views can be displayed using STIC, thus the echocardiographic protocol of the cardiovascular joint could be standardized. The reference range of the angle between ascending aorta and main pulmonary artery is clinically useful in prenatal screening of CTD and provides a reliable quantitative standard to estimate the spatial relationship of the large arteries of fetus.
